# Thirty Years of Nutrient Biogeochemistry in the Barents Sea and the adjoining Arctic Ocean, 1990–2019

**DOI:** 10.1038/s41597-022-01781-w

**Published:** 2022-10-22

**Authors:** Kjell Gundersen, Jane S. Møgster, Vidar S. Lien, Elizaveta Ershova, Linda F. Lunde, Hilde Arnesen, Ann-Kristin Olsen

**Affiliations:** 1 Plankton Research Group, Institute of Marine Research, P.O. Box 1870 Nordnes, NO-5817 Bergen, Norway; 2 Oceanography and Climate Research Group, Institute of Marine Research, P.O. Box 1870 Nordnes, NO-5817 Bergen, Norway

**Keywords:** Element cycles, Chemical ecology

## Abstract

This dataset contains biogeochemical samples from the Barents Sea and Arctic region analyzed by the Plankton Chemistry Laboratory at the Institute of Marine Research (IMR). Number of surveys and stations visited in the Barents Sea and Arctic has varied over the last 30 years. One major effort is the annual *Ecosystem Survey* in the fall, with multiple trawl surveys, net tows and CTD water sampling. Additionally, two transects are visited multiple times each year (Fugløya-Bjørnøya and Vardø-North). Only samples collected from water bottles are reported here. Bottle samples from each CTD cast were collected for dissolved inorganic nutrients (nitrate, nitrite, phosphate, silicate) and phytoplankton chlorophyll-*a* and phaeopigments (ChlA, PHAEO) at predetermined depths and for later analysis at IMR. On occasion, short-term projects have performed Winkler dissolved oxygen titrations (DOW) and particulate organic carbon and nitrogen (POC, PN) determinations. This unique data set has seen limited use over the years but is a great contribution towards global ocean research and climate change investigations.

## Abstract (summary)

**Table:** 

Measurement(s)	dissolved inorganic nutrients (nitrate, nitrite, phosphate, silicate) • cellular pigments (chlorophyll-a, phaeopigments) • dioxygen dissolved in marine water • particulate organic carbon and nitrogen
Technology Type(s)	nutrients autoanalyser • fluorometer • Winkler titration • CHN analyzer
Factor Type(s)	nitrate • nitrite • phosphate • silicate • chlorophyll-a • phaeopigment • dissolved oxygen • particulate organic carbon • particulate nitrogen
Sample Characteristic - Organism	phytoplankton
Sample Characteristic - Environment	sea water
Sample Characteristic - Location	Barents Sea and Arctic Ocean

## Background & Summary

The failing capelin fisheries in the Barents Sea initiated the *Open Ocean Time-series Program* (Havovervåkingen) at IMR in the late nineteen eighties, starting with a number of large-scale short-term surveys in the Barents Sea region. *PRO MARE* and other shorter-term projects had demonstrated the significance of seasonal cycling of phytoplankton blooms to zooplankton biomass, and how critical these sources of food can be for fish larvae^1^. In 1990, the *MARE NOR* program was launched to forecast marine resource developments in order to improve fisheries management^1^. In 2003, annual joint Russian-Norwegian fisheries surveys were combined into one investigation and became the annual *Ecosystem Survey* of the Barents Sea^2^.

**Fig. 1:**
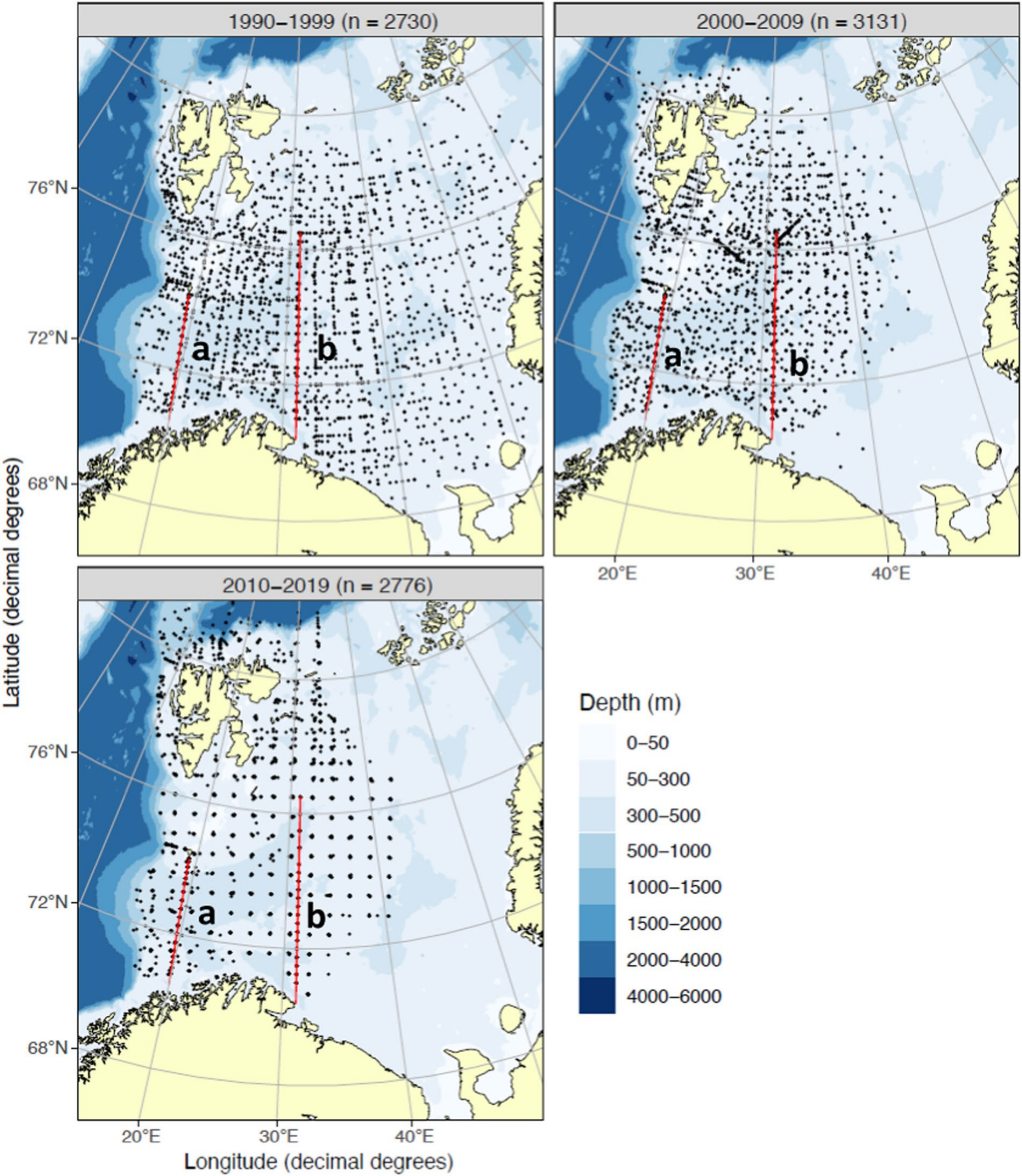
Distribution of sampling stations over three decades in the Barents Sea and the adjoining Arctic Ocean (closed circles). The red lines show the two seasonal transects in the *Open Ocean Monitoring Survey* (a = Fugløya-Bjørnøya; b = Vardø-North) and total number of stations visited (n) is summarized for each decade.

The number of surveys and stations visited annually in the Barents Sea has varied over the 30 years reported here (Fig. 1). To this day, the annual *Ecosystem Survey* is a Russian-Norwegian effort, and 2–3 research vessels from IMR will cover the entire Norwegian side of the Barents Sea with trawl surveys, net tows and CTD water sampling. As the summer ice edge has receded further north in recent years, the continental slope to the Polar Basin (north of the Barents Sea) has been added to these surveys. In addition to the annual *Ecosystem Survey* two trans-sectional core surveys, the Fugløya-Bjørnøya and the Vardø-Nord transects (Fig. 1), are still conducted 4–6 times each year. Water samples are also collected on short-term sampling projects (ships of opportunity) by all major ships operated by the IMR (Table 1). On these cruises, each CTD cast is sampled for dissolved inorganic nutrients (nitrate, nitrite, phosphate, silicate) and phytoplankton pigments (chlorophyll-*a*, phaeopigments) at predetermined depths (Table 2) for later analysis. Occasionally, short-term projects have also performed Winkler DO titrations (DOW) and particulate organic carbon and nitrogen (POC, PN) determinations. This set of quality-controlled data, from 30 years of ocean surveys conducted by the IMR, has never been published in its entirety.

**Table 1:** Ships number codes (in numerical order) of Norwegian research vessels used to collect biogeochemical samples between 1990–2019.

Ships number code	Name of research vessel	Ownership
1002	R/V Haakon Mosby	IMR
1003	R/V Fridtjof Nansen	IMR
1006	R/V R.U. Sverdrup	UiB
1007	R/V R.U. Sverdrup	UiB
1010	R/V Johan Ruud	UiO
1011	R/V Trygve Braarud	UiT
1015	R/V Dr. Fridtjof Nansen	NORAD
1017	R/V Michael Sars	IMR
1018	R/V Johan Hjort (until 1981)	IMR
1019	R/V Johan Hjort (from 1982)	IMR
1021	M/S Peder Roennestad	Hired for IMR surveys
1022	M/S Eldjarn	Hired for IMR surveys
1024	R/V G.O. Sars (until 2002)	IMR
1025	R/V G.M. Dannevig (until 1985)	IMR
1026	R/V G.M. Dannevig (from 1986)	IMR
1031	M/S Fjordfangst	Hired for IMR surveys
1043	M/S Opal (1986–1991)	Hired for IMR surveys
1044	R/V Lance	UiT
1047	M/S Endre Dyrøy	Hired for IMR surveys
1150	R/V Polarfront	OWM, MET
1170	R/V Hans Brattstrøm	UiB
1171	R/V Braarud	UiO
1173	R/V Jan Mayen (Helmer-Hanssen)	UiT
1187	K/V Andenes	KNM
1188	K/V Senja	KNM
1189	K/V Nordkapp	KNM
2704	M/S Christine E	Hired for IMR surveys
3005	M/S Nordengen	Hired for IMR surveys
3381	M/S Eros	Hired for IMR surveys
4135	M/S Nybo	Hired for IMR surveys
4174	R/V G.O. Sars (from 2003)	IMR
9999	Unknown	Hired for IMR surveys

The overview also details hired vessels and actual ownership by; Institute of Marine Research (IMR); University of Bergen (UiB); University of Oslo (UiO); University of Tromsø (UiT); Norwegian Agency for Development Cooperation, Oslo (NORAD); The Institute of Meteorology, Oslo (MET) with Ocean Weather Station Mike (OWM); and The Royal Norwegian Navy, Bergen (KNM; Kongelig Norsk Marine).

**Table 2:** A guide to standardized sampling depth intervals of CTD stations at various depths (designated bottom line in table).

	1	2	3	4	Water bottle number			8	9	10	11	12
					5	6	7					
Sample depths (m)	50	30	20	10	5							
	75	50	30	20	10	5						
	100	75	50	30	20	10	5					
	125	100	75	50	30	20	10	5				
	150	125	100	75	50	30	20	10	5			
	200	150	125	100	75	50	30	20	10	5		
	250	200	150	125	100	75	50	30	20	10	5	
	300	250	200	150	125	100	75	50	30	20	10	5
	400	300	250	200	150	100	75	50	30	20	10	5
	500	400	300	200	150	100	75	50	30	20	10	5
	600	500	400	300	200	150	100	75	50	30	10	5
	800	600	500	400	300	200	150	100	75	50	30	10
	1000	800	500	400	300	200	150	100	75	50	30	10
	1200	1000	800	500	400	300	200	150	100	50	30	10
	1500	1200	1000	800	500	400	300	200	100	50	30	10
	1800	1500	1200	1000	800	500	400	200	100	50	30	10
	2000	1800	1500	1000	800	500	400	200	100	50	30	10
	2500	2000	1500	1000	800	500	400	200	100	50	30	10
	3000*	2500*	2000*	1500*	1000*	800*	500*	200*	100*	50*	30*	10*

*Nominal station depth (m).

Nutrient samples (nitrate, nitrite, phosphate, silicate) are collected from the maximum number of depths at each CTD station^12^, while the number of Chlorophyll-a samples (ChlA) are only collected in the upper 100 m of the water column. On dedicated cruises, Winkler DO (DOW), and particulate organic carbon and nitrogen (POC, PN) samples were collected from random depths in the upper 150 m of the water column (specific depths are not shown here).

## Methods

### Sample collection and analysis

Seawater samples are collected from Niskin-type water bottles at predetermined depths (Table 2) triggered by a CTD (Conductivity Temperature Depth) unit mounted on a rosette. Two types of CTD have been used on our survey ships over the years (Table 3); the Neil-Brown MK3B and MK3C systems (Commonwealth Scientific and Industrial Research Organisation [CSIRO], Division of Marine Research, Australia) were most common up until the millennium, when it was replaced with Sea-Bird Scientific (SBE) 911plus units (Sea-Bird Scientific, USA). Total numbers of stations sampled has varied over the years but IMR has, in addition to more random visits to the region on short-term projects, been visiting each of the two transects approximately 4–5 times each year (Fig. 1). Additionally, an annual, full-scale survey covering major parts of the Barents Sea has been conducted each year since 2003, usually in concert with one or two other IMR ships and a Russian ship covering the Russian sector.

**Table 3:** Research ships (and year built) at IMR between 1990–2019, equipped with a Neil-Brown or a SeaBird Electronics CTD rosette.

Research ship	CTD	
R/V Michael Sars (1979)	N-B (1990–2002)	
R/V Håkon Mosby (1980)	N-B (1990–2002)	SBE (2003–2015)
R/V Johan Hjort (1990)	N-B (1990–1997)	SBE (1998-today)
R/V G.O. Sars (2003)	N-B (1990–1998)	SBE (1999-today)
R/V Kristine Bonnevie (2016)	SBE (2016-today)	

### Dissolved inorganic nutrients (nitrite, nitrate, phosphate, silicate)

After three rinses, each water sample (20 mL) was collected in a polyethylene vial. Up until the turn of the century (1999–2002) most nutrient samples were analysed in real time onboard the research vessels and without chloroform additions. As the research cruise activity expanded, the number of automated analysers could no longer match the number of ships operating simultaneously (Table 3) and nutrient samples were added chloroform (200 µL) to retard biological activity and stored at + 4 °C for analysis in the home laboratory, usually within 1–6 weeks. Prior to analysis, the samples were acclimated to room temperature as the evaporating chloroform was evacuated by vacuum prior to analysis on an Automated Analysis (AA) system. A number of automated systems have been in use over the years (Table 4) and up until recently, all nutrients were run on homemade Skalar and Alpkem hybrid systems. The latest upgrade was in 2017 when a complete AA system was purchased from Skalar Analytical B.V. (The Netherlands) for the first time. Colorimetric determinations of dissolved inorganic nutrients are based on the methods first described by Bendschneider & Robinson^3^ and Grasshof^4^ with a number of minor adjustments suggested by the manufacturers (Alpkem, Skalar). The AA system measures nitrate, nitrite, phosphate and silicate. Briefly, nitrate in seawater is reduced to nitrite coupled to a diazonium ion and, in the presence of aromatic amines, the resulting blue azo-dye is determined spectrophotometrically at 540 nm. The nitrate concentration is corrected for ambient nitrite (same analytical method as for nitrate, but without cadmium reduction) measured concurrently. Phosphate reacts with molybdate at low pH and the resulting phosphomolybdate is reduced with ascorbic acid to a blue complex measured spectrophotometrically at 810 nm. With the new Skalar AA purchased in 2017 (Table 4) the phosphomolybdate peak is now measured at 880 nm. Silicate (silicic acid) is reacting to molybdate at low pH and the resulting silicomolybdate is reduced by ascorbic acid to a blue dye measured spectrophotometrically at 810 nm.

**Table 4:** Use of analytical instruments (1990–2019) in the Plankton Chemistry Laboratory showing analytical range, precision and accuracy.

Instrument	Lab. reg.	Years	Parameter	Range	Precision (%)	Accuracy (%)
Skalar-hybrid (Skalar Instruments)	Skalar-A	1983–2001	Nitrate	0.5–20 µM	<0.2	<1
			Nitrite	0.05–2.5 µM	<0.2	<1
			Phosphate	0.02–1.5 µM	<1	<2
			Silicate	0.15–20 µM	<0.2	<1
Skalar-hybrid (Skalar Instruments)	Skalar-B	1978–2008	Nitrate	0.5–20 µM	<0.2	<1
			Nitrite	0.05–2.5 µM	<0.2	<1
			Phosphate	0.02–1.5 µM	<1	<2
			Silicate	0.15–20 µM	<0.2	<1
Akpkem (O.I. Analytical)	Alpkem-C	2004–2014	Nitrate	0.5–20 µM	<0.2	<1
			Nitrite	0.05–3 µM	<0.2	<1
			Phosphate	0.06–3 µM	<2	<2
			Silicate	0.4–20 µM	<0.2	<1
Skalar-hybrid (Skalar Instruments)	Skalar-D	2008–2017	Nitrate	0.4–20 µM	<0.2	<1
			Nitrite	0.06–3 µM	<0.2	<1
			Phosphate	0.06–3 µM	<2	<2
			Silicate	0.7–20 µM	<0.2	<1
Skalar San++ Continuous Flow Analyzer	Skalar-F	2017-present	Nitrate	0.5–50 µM	<0.2	<1
			Nitrite	0.06–5 µM	<0.2	<1
			Phosphate	0.06–5 µM	<1	<2
			Silicate	0.7–150 µM	<0.2	<1
Turner Design 10 AU	Turner	1990-present	ChlA	0.005–0.25 mg/m3	<1	<3
			Phaeo	0.005–0.25 mg/m3	<1	<3
Carlo-Erba 1106 Strumentazione	Carlo-Erba	1990–2003	POC	0.004–0.7 mg	> 1	<1
			PN	0.001–0.12 mg	<1.5	<1
Thermo Finnegan Flash EA1112	Finnegan	2004–2016	POC	0.004–0.7 mg	<1	<1
			PN	0.001–0.12 mg	<1	<1
rapid MICRO N cube(*)	Elemental	2017-present	POC	0.004–1.2 mg	<1	<1
			PN	0.001–s0.2 mg	<1	<1
916 Ti-Touch	Winkler-DO	2019-present	DOW	0.06–90 mL/L	<0.002	NA
916 Ti-Touch	Winkler-DO	2020-present	DOW	0.06–90 mL/L	<0.002	NA
Titrino 665	Dozimat	2000-present	DOW	0.08–90 mL/L	<0.002	NA

Analyzed ChlA and Phaeo shows measuring range in acetone extracted solution, and POC and PN shows analytical range in a filter-collected sample.

(*) = originally designed for N-analysis only; in 2020 the intrument was retrofitted to inlcude C-analysis and renamed *UNICUBE trace*.

### Chlorophyll-a and phaeopigment samples (ChlA, Phaeo)

A standard volume (265 mL) is collected from each depth (Table 2), filtered onto a 25 mm GFF filter and stored at −20 °C until analysis in the land-based laboratory. Historically, pigment samples were transported home by one of the cruise participants, as hand-luggage in a cooler with frozen cooler-elements. These days, pigment samples are brought back in specially designed coolers, with an internal temperature recorder, that is rated for −20 °C for a minimum of 3 days. In the laboratory, the samples are thawed in 90% acetone, and stored at +4 °C overnight before analysis on a Turner Design 10 AU fluorometer. Phaeopigments (Phaeo) are measured separately from ChlA, in a second reading of the sample after adding 3 drops of a weak acid (5% HCl). The fluorometer is standardized regularly using a solid standard with known fluorescence, and in accordance with Holm-Hansen & Riemann^5^ and the manufacturer^6^. Up until the 2008 the drift in the light-source was monitored annually but from 2009 the solid standard has been recorded every time the fluorometer is used.

### Particulate organic carbon and nitrogen (POC, PN) samples

A standard volume (265 mL) is collected from each depth (Table 2) and filtered onto a pre-combusted 25 mm GFF filter (+450 °C, min. 4 h). Each sample is stored frozen (−20 °C) in a pre-combusted glass tube until analysis in the land-based laboratory. Preparations of samples and analysis of elemental C and N is described in detail in Grasshof *et al*.^7^. Briefly, the dried filter-samples are fumed in acid (conc. HCl) in a desiccator for 4–12 h, before they are dried again and packed in a tin-foil capsule. Analysis of POC and PN is performed on an elemental analyzer (see Table 4 for details) and in accordance with the manufacturer’s recommendations.

### Dissolved oxygen samples by winkler titrations (DOW)

Samples for dissolved oxygen are collected in volume-calibrated glass BOD bottles (approximate volume 125 mL) and filled from bottom up using a Tygon-tubing. The sample is let to overflow approximately 3 times the volume and great care is taken to avoid small air bubbles at the inside of the sample bottle. Thiosulfate titrations of dissolved oxygen are still done as first described by Winkler^8^ but the method has seen some updates and improvements over the years^9–11^. Grasshof *et al*.^7^ is describing in detail the current method of sample collection, pretreatment and coulometric titrations of Winkler samples. Briefly, dissolved oxygen is reacting with an alkaline solution (Reagent 1) to form a manganese-hydroxy-complex. Under alkaline conditions, the Mn-complex is reacting with the iodide solution (Reagent 2) and let to precipitate at the bottom of the sample bottle. The sample is added 10 N sulphuric acid to dissolve the iodide precipitate (pH = 1–2.5) and the yellow iodine is titrated by thiosulfate to a clear solution. The titrant is standardized by a known concentration of potassium iodate (KIO3) as described by Grasshof *et al*.^7^.

## Data Records

Data from the Barents Sea region and the adjoining Arctic Ocean has been submitted to the SeaDataNet (SDN) services (https://www.seadatanet.org/), and can be downloaded as a 1990–2019 compilation^12^. SeaDataNet is the standard publication format for biogeochemical data at IMR and the specifics of the data file format chosen here (netCDF) can be found in chapter 4.5.1 of the current version (v.1.22) at https://archimer.ifremer.fr/doc/00454/56547/. Key features of SeaDataNet include rigorous use of the Natural Environment Research Council (NERC, UK) libraries, including measurement techniques (P01) and use of data units (P06). The file format is self-contained for both datasets and variables, and we have chosen variable names most familiar to the ocean science community. All data in this paper are considered public domain and, as such, are also integrated with the Copernicus Marine Service (https://marine.copernicus.eu/) and the EMODnet Chemistry (https://www.emodnet-chemistry.eu/) platforms.

**Fig. 2:**
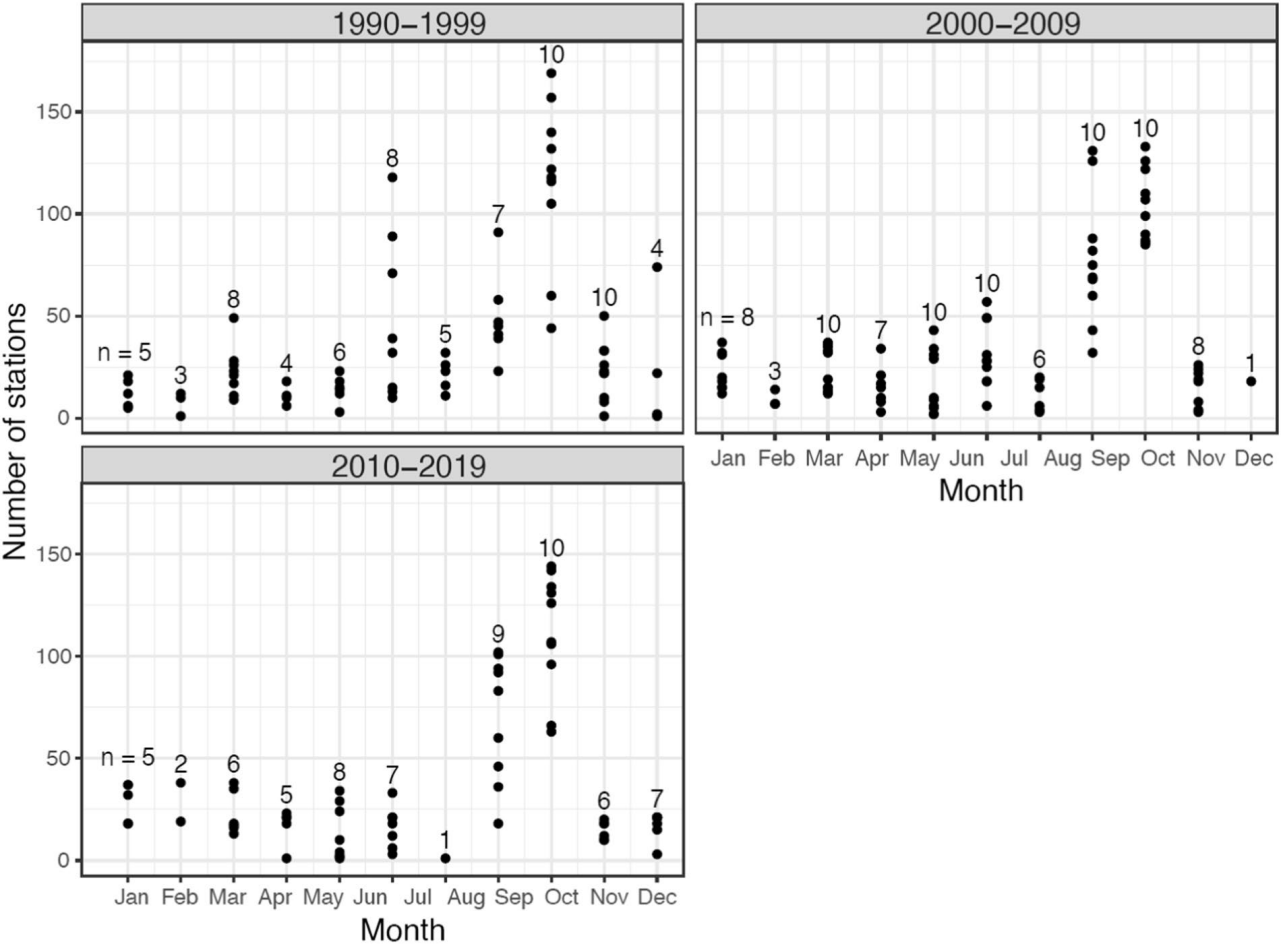
Number of stations visited over the course of a year, during three decades in the Barents Sea and the Arctic Ocean (1990–2019). The number above each column (n) shows total number of years that were sampled during that month, in each decade.

Distribution and number of stations visited has changed over the three decades presented here (Fig. 1). The first decade (1990–1999) had sampling stations more scattered and collections were made both in Norwegian and Russian sector of the Barents Sea region (total number of stations = 2730). The following decade (2000–2009) saw more stations sampled (total number = 3131), but largely in the Norwegian sector of the Barents Sea. Due to the receding ice during summer, this is also the decade where increasing number of stations were sampled north of Svalbard, in the adjoining Arctic Ocean (Fig. 1). During the last decade (2010–2019) number of stations are similar to 1990–1999 levels (total number = 2776) but the exact same station locations are sampled repeatedly each year, and mostly in the Norwegian sector. Distribution of stations visited within a year (Fig. 2) shows a maximum coverage in August-September during all three decades. Each month is not always visited each year (n < 10 in Fig. 2) but September, the month of the *Ecosystem Survey*, shows maximum number of stations that has been sampled each year through all three decades (n = 10).

**Fig. 3:**
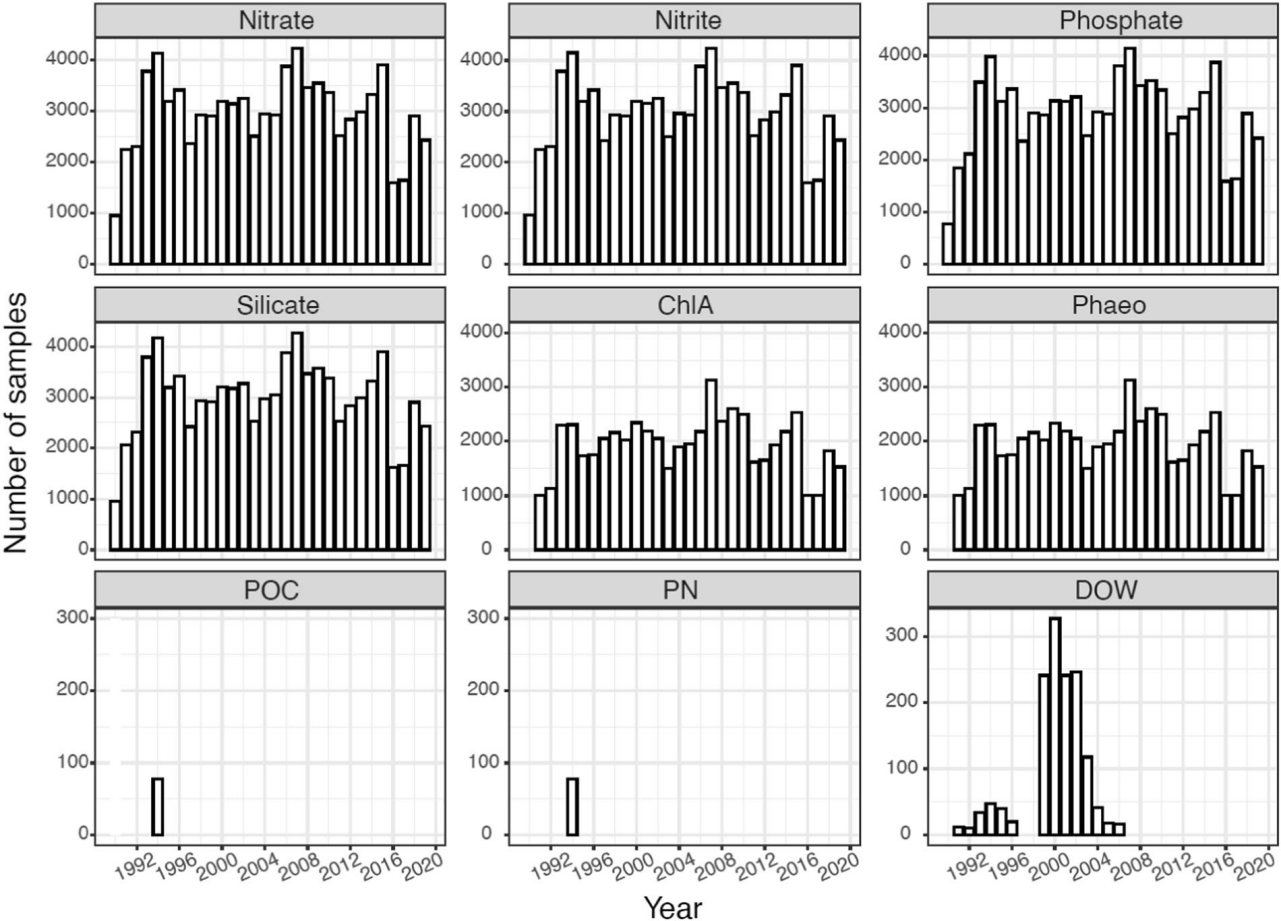
Number of samples collected each year, from the Norwegian, Greenland and Iceland Seas. Note the different scale on the y-axis between nutrients (nitrate, nitrite, phosphate, silicate), pigments (ChlA, Phaeo) and dissolved oxygen (DOW), and particulate C and N (POC, PN).

This data set (Fig. 3) contains nutrient measurements (nitrate, nitrite, phosphate and silicate), pigments (ChlA and Phaeo), particulate organic carbon and nitrogen (POC, PN), and Winkler titrations of dissolved oxygen (DOW). Peak number of samples range between 3000–4000 for nutrients and 2000–3000 pigment samples analyzed each year (Fig. 3). The lowest number of samples were collected at the very beginning of the times-series, and during selected years (1996–1997, 2002–2004, 2010–2012, 2015–2017) associated with reduced number of cruises to the region (Fig. 3). Only on one cruise were POC and PN samples collected from the region (Fig. 3) and only on a minor number cruises were DOW measured by Winkler titrations. All data were quality controlled (QC) by analysts using quality flags 0–5 (Table 5) in accordance with Jaccard *et al*.^13^ and the *OceanSITES Data Format Reference Manual V.1.4* (http://www.oceansites.org/docs/oceansites_data_format_reference_manual.pdf), and is available at the *Norwegian Marine Data Center* (NMDC) home page at IMR (http://metadata.nmdc.no/metadata-api/landingpage/b6ce5b610e4ba316bce2cae9ec2aed61).

**Table 5:** Quality control (QC) flags used for dissolved inorganic nutrients (nitrite, nitrate, phosphate, silicate), pigments (ChlA, Phaeo), particulates (POC, PN) and Winkler dissolved oxygen (DOW) samples.

ASCII flag	netCDF flag	Action
0	[48, or 48b]	No QC performed
1	[49, or 49b]	Good data
2	[50, or 50b]	Data did not conform to expected outcome, but cannot be discarded
3	[51, or 51b]	Data appears compromised and cannot be corrected
4	[52, or 52b]	Bad data clearly beyond correction
5	[53, or 53b]	Good data that appears misplaced, and has been corrected
6	[54, or 54b]	Data value below detection
7	[55, or 55b]	Measured value beyond detection
8	[56, or 56b]	Interpolated value
9	[57, or 57b]	Missing data
A	[65, or 65b]	Uncertainty of the data value

The dataset is distributed in two file formats; a comma separated values (ASCII format) or a network common data form (NetCDF). Selected data will therefore appear in the ASCII format with numeral flags (ASCII flag), or as 8-byte numerals in the netCDF format (netCDF flag). See Jaccard *et al*.^13^ for more detailed descriptions of the terms used below. The data header will list all the flags listed, but only QC flags 0–5 (ASCII) and flags 48–53 or 48b–53b (netCDF) were used to evaluate our data.

Only QC flags 1, 2, and 5 are available from these data records. “Good data” (flag = 1) are data that passed QC, “probably good data” (flag = 2) are data that may seem unexpected but (based on information from sampling and analysis) we have no reason to exclude them; “value changed” data (flag = 5) are obvious errors based on notes from the sample sheet, where analytically correct data has been relocated to another depth (e.g. where the entire nutrient profile has been mislabelled and logged in “up-side-down”). In the cases where single samples appear mislabelled, exhibit analytical errors, or appear to fall outside expected QC envelope, such values are discarded as “bad data” (flags = 3 and 4 are dedicated to doubtful and erroneous data, respectively). All data (Table 6) are hosted on an *OPeNDAP* server at IMR and available both as netCDF4 and csv files. The netCDF file (Table 7) follow the *SeaDataNet* format for profile data (https://www.seadatanet.org/Standards/Data-Transport-Formats).

**Table 6:** Groups of parameters as they appear in netCDF with method descriptions (P01 strings) as listed by the National Environmental Research Council, the NERC Vocabulary Server (NVS) hosted by the British Oceanographic Data Centre BODC (http://vocab.nerc.ac.uk/search_nvs/).

Parameter	This study	BODC concept ID	Parameter description	Approved BODC units		CMEMS	Norwegian Water Frame Directive	
	Acronym	SDN:P01		SDN:P06	SDN:P06	Acronym	Parameter (Norw.)	Acronym (Norw.)
Nitrate	Nitrate	CHEMM012	Concentration of nitrate per unit volume of water (dissolved plus reactive particulate phase) with a correction for nitrite	UPOX	umol/L	NTRA	Nitrat	N-NO3
Nitrite	Nitrite	NTRIAAZX	Concentration of nitrite per unit volume of water (unknown phase)	UPOX	umol/L	NTRI	Nitritt	N-NO2
Phosphate	Phosphate	PHOSAATX	Concentration of phosphate per unit volume of water (dissolved plus reactive particulate phase)	UPOX	umol/L	PHOS	Fosfat	P-PO4
Silicate	Silicate	SLCAAATX	Concentration of silicate per unit volume of water (dissolved plus reactive particulate phase)	UPOX	umol/L	SLCA	Silikat	SI-SIO2
Chlorophyll-a	ChlA	CPHLFLP1	Concentration of chlorophyll-a per unit volume of water (particulate >GF/F phase)	UMMC	mg/m3	CPHL	Klorofyll-a	KLFA
Phaeopigments	Phaeo	PHAEFLP1	Concentration of phaeopigments per unit volume of water (particulate >GF/F phase)	UMMC	mg/m3	PHAEO	Feopigmenter	Feo
Particulate organic C	POC	CORGCAP1	Concentration of organic carbon per unit volume of water (particulate >GF/F phase)	UPOX	umol/L	POC	Partikulært organisk karbon	POC
Particulate N	PN	NTOTCAP1	Concentration of total nitrogen per unit volume of water (particulate >GF/F phase)	UPOX	umol/L	PN	Partikulært organisk nitrogen	PON
Winkler dissolved oxygen	DOW	DOXYWITX	Concentration of oxygen per unit volume of the water body [dissolved plus reactive particulate phase] by Winkler titration	UMLL	mL/L	DOX1	Winkler oksygen	O2

Associated units (P06) and abbreviations applied for this data set, the Copernicus Marine Service (CMEMS) abbreviations applied for this data set are shown, including acronyms used by the Copernicus Marine Service (CMEMS) and the Norwegian Water Frame Directive (Vanndirektivet).

**Table 7:** Example of netCDF file extracted from SeaDataNet (SDN), showing cruise number (SDN_CRUISE), sampling station (SDN_STATION), position (Latitude, Longitude) and measured bottom depth using an ecco sounder (SDN_BOT_DEPTH).

Time	SDN_CRUISE	SDN_STATION	Latitude	Longitude	SDN_BOT_DEPTH	PRES	depth	Nitrate	Nitrite	Phosphate	Silicate	ChlA	Phaeo	POC	PN	DOW
UTC			degrees_north	degrees_east	m	dbar	m	umol L-1	umol L-1	umol L-1	umol L-1	mg m-3	mg m-3	umol L-1	umol L-1	mL L-1
1994-06-09T10:09:00Z	1994207	374	73.17	25.22	405	0	0	2.84	0.03	0.39	3.14	0.53	0.09	NaN	NaN	NaN
1994-06-09T10:09:00Z	1994207	374	73.17	25.22	405	5	5	3.06	0.08	0.44	3.18	0.54	0.10	2.92	0.50	NaN
1994-06-09T10:09:00Z	1994207	374	73.17	25.22	405	10	10	3.33	0.09	0.45	3.63	0.43	0.07	NaN	NaN	NaN
1994-06-09T10:09:00Z	1994207	374	73.17	25.22	405	20	20	3.18	0.09	0.45	3.18	0.46	0.11	4.08	0.64	NaN
1994-06-09T10:09:00Z	1994207	374	73.17	25.22	405	30	30	6.24	0.12	0.65	4.47	0.09	0.03	NaN	NaN	NaN
1994-06-09T10:09:00Z	1994207	374	73.17	25.22	405	50	50	9.78	0.17	0.84	5.00	0.01	0.08	1.42	0.21	NaN
1994-06-09T10:09:00Z	1994207	374	73.17	25.22	405	75	75	11.92	0.11	0.89	5.27	0.02	0.06	NaN	NaN	NaN
1994-06-09T10:09:00Z	1994207	374	73.17	25.22	405	100	100	11.72	0.13	0.88	5.29	0.00	0.07	1.17	0.14	NaN
1994-06-09T10:09:00Z	1994207	374	73.17	25.22	405	150	150	10.72	0.20	0.86	5.26	0.01	0.10	NaN	NaN	NaN
1994-06-09T10:09:00Z	1994207	374	73.17	25.22	405	200	200	11.05	0.29	0.87	5.36	0.01	0.05	2.08	0.29	NaN
1994-06-09T10:09:00Z	1994207	374	73.17	25.22	405	300	300	11.04	0.19	0.85	5.35	NaN	NaN	NaN	NaN	NaN
1994-06-09T10:09:00Z	1994207	374	73.17	25.22	405	394	394	11.87	0.08	0.94	6.28	NaN	NaN	2.17	0.29	NaN
1994-06-09T10:09:00Z	1994207	374	73.17	25.22	405	NaN	NaN	NaN	NaN	NaN	NaN	NaN	NaN	NaN	NaN	NaN
1994-06-09T10:09:00Z	1994207	374	73.17	25.22	405	NaN	NaN	NaN	NaN	NaN	NaN	NaN	NaN	NaN	NaN	NaN
1994-06-09T10:09:00Z	1994207	374	73.17	25.22	405	NaN	NaN	NaN	NaN	NaN	NaN	NaN	NaN	NaN	NaN	NaN
1994-06-09T10:09:00Z	1994207	374	73.17	25.22	405	NaN	NaN	NaN	NaN	NaN	NaN	NaN	NaN	NaN	NaN	NaN
1994-06-09T10:09:00Z	1994207	374	73.17	25.22	405	NaN	NaN	NaN	NaN	NaN	NaN	NaN	NaN	NaN	NaN	NaN
1994-06-09T10:09:00Z	1994207	374	73.17	25.22	405	NaN	NaN	NaN	NaN	NaN	NaN	NaN	NaN	NaN	NaN	NaN
1994-06-09T10:09:00Z	1994207	374	73.17	25.22	405	NaN	NaN	NaN	NaN	NaN	NaN	NaN	NaN	NaN	NaN	NaN
1994-06-09T10:09:00Z	1994207	374	73.17	25.22	405	NaN	NaN	NaN	NaN	NaN	NaN	NaN	NaN	NaN	NaN	NaN
1994-06-09T10:09:00Z	1994207	374	73.17	25.22	405	NaN	NaN	NaN	NaN	NaN	NaN	NaN	NaN	NaN	NaN	NaN
1994-06-09T10:09:00Z	1994207	374	73.17	25.22	405	NaN	NaN	NaN	NaN	NaN	NaN	NaN	NaN	NaN	NaN	NaN

Sampling depths are displayed as original pressure readings at the time of collection (PRES) and recalculated to meters (depth) using CTD temperature and density. Dissolved inorganic nutrients (Nitrate, Nitrite, Phosphate, Silicate), pigment content (ChlA, Phaeo), particulate organic C and N (POC, PN) and Winkler titrations of dissolved oxygen (DOW), are shown where samples have been collected and depths without sample collections are denominated ‘not a number’ (NaN). For brevity, columns with quality control (QC) flags, associated with each measured parameter (parameter_SEADATANET_QC) are not included in this overview.

## Technical Validation

The Plankton Chemistry Laboratory at IMR maintain quality control of precision and accuracy by daily assessments of analytical standard curves of internal standards. As large data sets are becoming increasingly available for a wider, global array of users, it is important that the scientific community agree on a set of common practices that will lead to a common QC. Therefore, our laboratory find it crucial to maintain contacts with other laboratories through regional intercalibration studies such as *QUASIMEME* (http://www.quasimeme.org/) and in global intercalibrations such as IOCCP (https://repository.oceanbestpractices.org/handle/11329/883).

Our 30-year time-series of seawater nutrients are one of the most complete sets of data to date. However, handling and storage of, for instance, nutrient samples have varied considerably over this time as in the rest of the scientific community (see recent overview by 14). Early in our time-series (the first decade) seawater nutrients were commonly analyzed at sea using an onboard, automated nutrient analyzer. There are considerable challenges to maintaining high quality and analytical precision on a moving ship at sea, as well as the need for qualified people to follow the ship on all cruises. During the last decades the number of samples analyzed by the Plankton Chemistry Laboratory, and the number of vessels used simultaneously in different oceans, have increased dramatically. Therefore, we are currently analyzing a total of 70,000–110,000 nutrient samples each year. Mostly due to these cost-effective and practical considerations two decades ago, the laboratory decided to continue with one single, land-based analytical unit with high throughput of samples poisoned with chloroform. The laboratory-based unit is manned by highly skilled analysts that can take turns and work uninterrupted and continuously over long periods of time. This arrangement also means that samples are now stored at sea for periods of time and transported to the land-based laboratory prior to analysis (see Storage of nutrient-samples below). Filter-collection and storage of frozen particulate samples (ChlA, Phaeo, POC, PN), and onboard Winkler titrations of DOW samples, do follow internationally recognized guidelines for time-series measurements (e.g.^11,14^).

### Storage of nutrient-samples

Our collection of unfiltered seawater samples, added small aliquots of chloroform, is not commonplace in ocean research and the choice of various storage techniques have been debated for decades^15^. However, when ship-based analysis is no longer an option (see section above), there is a need to poison or preserve the samples prior to analysis. Adding preserving agents may contaminate the samples, so great care must be taken and only analytical grade additives can be used for this purpose. If you chose to prefilter the samples before storing them frozen at −20 °C, on the other hand, the very act of prefiltration may also be a source of contamination^14^. An added drawback with frozen samples, is that recovery after thawing is not always complete at high silicate concentrations^16^. Therefore, the Plankton Chemistry Laboratory decided early on to poison the nutrient samples with analytical grade chloroform and store them at +4 °C until analysis (see Methods for details). Our nutrient samples are collected in 20 mL polyethylene vials (PE scintillation vials), added 200 µL chloroform to prevent further biological activity, and stored (up until 3 months) in the dark at +4 °C until analysis. The PE vials are used directly from the package without being acid-washed. Initially, we had some concerns that the PE vials would contaminate the nutrient samples. However, several comparative studies, between acid-washed and unwashed PE-bottles, did not show any discernable difference for any of the 4 nutrients (data not shown here).

**Fig. 4:**
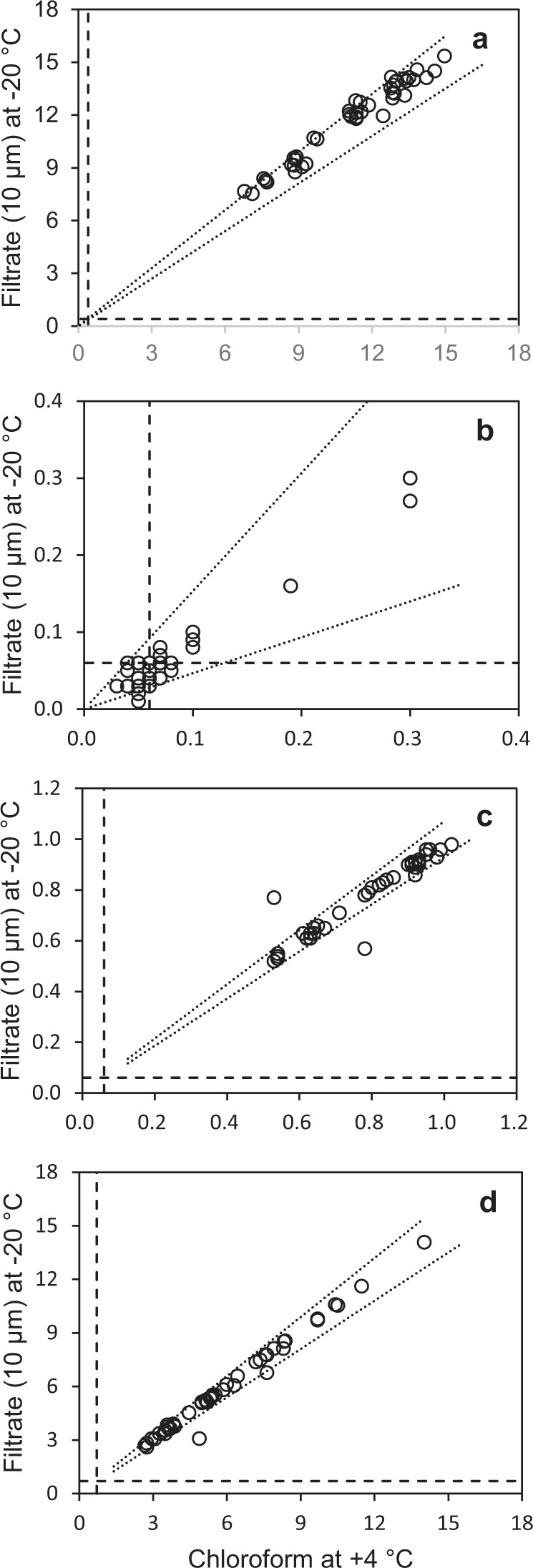
Comparison of nutrient samples that were filtered and stored at −20 °C for 3 months, and natural seawater samples added chloroform and stored equally long at +4 °C. Each panel shows lower level of detection for Skalar-D (Table 4) used for these analyses (horizontal and vertical broken lines) and estimated analytical error (dotted lines) associated with nitrate (**a**), nitrite (**b**), phosphate (**c**) and silicate (**d**) measurements.

We also had some concerns that the biological inhibitor (chloroform) could disrupt microplankton cells and add intracellular nutrients to the pool of inorganic nutrients in a sample vial, so we compared filtered and unfiltered nutrient samples. It is however more commonplace, as in large scale open ocean time-series programs, to store pre-filtered nutrient samples frozen^14,15^. Therefore, we combined these two issues and compared the two treatments; unfiltered seawater samples added chloroform and stored at + 4 °C (our standard mode of sampling and storage), and filtered seawater samples stored frozen at −20 °C. We used a 10 µm nylon filter disc since gravitational filtration did not work efficiently on smaller pore sizes in our samples from the Norwegian Sea. Nitrate (Fig. 4a) did show slightly elevated concentrations in the filtered samples stored frozen, but we found no difference between levels of nitrite, phosphate, and silicate in the two treatments (Fig. 4b–d). The difference between frozen and chloroform treated samples analyzed for nitrate however, was not beyond analytical error (10%) for this analysis (Fig. 4a). Our findings are comparable to Dore *et al*.^16^, who found no difference between frozen samples and natural seawater that was analyzed immediately in a lower range of nutrient concentrations from the Pacific Ocean, and we concluded that samples added chloroform and stored at + 4 °C worked equally well to frozen samples.

## Usage Notes

All data in this publication are available in accordance with *Creative Commons* rules (https://creativecommons.org/licenses/by/4.0/) and users are free to share, copy and redistribute the material in any medium or format. The data can be adapted and built upon at users own volition as long as the data source is appropriately accredited.

Quality control of large scale, long-term data sets are crucial to account for potential mislabeling, potential errors in storage and handling of the samples leading to contamination, and potential anomalies during analysis of the samples. The Plankton Chemistry Laboratory is currently using a QC-flagging system to account for the quality of the data that is produced (Table 5). Data with flags 1, 2 and 5 are made available for use in this publication, while flags 3 and 4 are deemed compromised and are not included. Due to the many different people participating in the sampling program on IMR cruises, we must accept that minor mistakes can be made. Some of these can be corrected (e.g. a mislabeled depths with a value that clearly belongs somewhere else) and are given Flag = 5, while others are beyond correction (e.g. samples that shows signs of contamination or appears to have been stored too long) and are given flags 3 or 4. An important QC flag is number 2, where we label data points that are outside expected value, but for no apparent reason. Flag number 2 data can be an expression of small term changes that may turn out to be significant over a larger time scales, and this kind of data cannot be discarded in ocean monitoring studies lasting decades to centuries. Unfortunately, data from samples collected prior to 2010 in our time-series used a different QC definition of flag number 2, and these data were excluded. Therefore, flags number 2, 3 and 4 were routinely excluded from data sets during the 1990–2009 period.

## Data Availability

No custom code was used to generate or process the data described in this paper.
